# Head to head evaluation of second generation ALK inhibitors brigatinib and alectinib as first-line treatment for ALK+ NSCLC using an *in silico* systems biology-based approach

**DOI:** 10.18632/oncotarget.27875

**Published:** 2021-02-16

**Authors:** Enric Carcereny, Alonso Fernández-Nistal, Araceli López, Carmen Montoto, Andrea Naves, Cristina Segú-Vergés, Mireia Coma, Guillem Jorba, Baldomero Oliva, Jose Manuel Mas

**Affiliations:** ^1^Catalan Institute of Oncology B-ARGO Group, Hospital Germans Trias i Pujol, Badalona, Spain; ^2^Takeda Farmacéutica España, Madrid, Spain; ^3^Anaxomics Biotech, Barcelona, Spain; ^4^Structural Bioinformatics (GRIB-IMIM), Departament de Ciències Experimentals i de la Salut, Universitat Pompeu Fabra, Barcelona, Spain

**Keywords:** ALK inhibitor, brigatinib, alectinib, mathematical model, systems biology

## Abstract

Around 3–7% of patients with non-small cell lung cancer (NSCLC), which represent 85% of diagnosed lung cancers, have a rearrangement in the ALK gene that produces an abnormal activity of the ALK protein cell signaling pathway. The developed ALK tyrosine kinase inhibitors (TKIs), such as crizotinib, ceritinib, alectinib, brigatinib and lorlatinb present good performance treating ALK+ NSCLC, although all patients invariably develop resistance due to ALK secondary mutations or bypass mechanisms. In the present study, we compare the potential differences between brigatinib and alectinib’s mechanisms of action as first-line treatment for ALK+ NSCLC in a systems biology-based *in silico* setting.

Therapeutic performance mapping system (TPMS) technology was used to characterize the mechanisms of action of brigatinib and alectinib and the impact of potential resistances and drug interferences with concomitant treatments.

The analyses indicate that brigatinib and alectinib affect cell growth, apoptosis and immune evasion through ALK inhibition. However, brigatinib seems to achieve a more diverse downstream effect due to a broader cancer-related kinase target spectrum. Brigatinib also shows a robust effect over invasiveness and central nervous system metastasis-related mechanisms, whereas alectinib seems to have a greater impact on the immune evasion mechanism.

Based on this *in silico* head to head study, we conclude that brigatinib shows a predicted efficacy similar to alectinib and could be a good candidate in a first-line setting against ALK+ NSCLC. Future investigation involving clinical studies will be needed to confirm these findings. These *in silico* systems biology-based models could be applied for exploring other unanswered questions.

## INTRODUCTION

Lung cancer (LC) remains the leading cause of death worldwide, with an estimated 1.6 million deaths each year [1, 2]. Despite significant therapeutic advances over the last decade, over half of patients diagnosed with LC die within one year of diagnosis and the five-year survival is around 18% [3]. About 85% of LCs are diagnosed as the subtype non-small cell lung cancer (NSCLC), adenocarcinoma being one of the most common histological subtypes. In adenocarcinoma, several driver mutations have been identified, including mutations/alterations of the epidermal growth factor receptor (EGFR), anaplastic lymphoma kinase (ALK), and ROS1, among others; most of them are therapeutically targetable [4]. Around 3–7% of NSCLC cases present active ALK rearrangement (ALK+ NSCLC) that produces an abnormal activity of the ALK protein cell signaling pathway and causes the cancer cells to grow and metastasize [5, 6]. Central nervous system (CNS) metastasis is a common finding in NSCLCs, occurring in 10% of patients, and even more frequent in ALK+ NSCLCs, were the frequency of CNS metastasis is around 20–30% at the time of diagnosis [7, 8]. CNS is also the most common site of relapse [9].

Thus far, three generations of ALK tyrosine kinase inhibitors (TKIs) have been developed. Some of the drugs that target the abnormal ALK protein are crizotinib (first generation), ceritinib, alectinib, brigatinib, ensartinib (second generation) and lorlatinib (third generation) [10]. However, despite their effectiveness in ALK+ NSCLC cases, all patients invariably develop treatment resistance at some point. Consequently, it is of the upmost importance to adequately use the currently available treatments in the correct order to maximise the life span of NSCLC patients. The most common progression mechanisms for all ALKi are: 1) ALK secondary mutations, which affect not only crizotinib-treated patients (around 20–30% of patients [11]), but also second and third generation ALKi [12, 13]; and 2) bypass mechanisms (i.e., activation of other parallel pro-proliferative signaling pathways [14]). Crizotinib has been used as first-line since its approval in 2011 in the United States [15] and in 2015 in Europe [11]. The results of its phase III trial (PROFILE 1014) demonstrated that crizotinib was superior to standard chemotherapy [16]. Second and third generation ALK TKIs are effective in treating numerous crizotinib-resistant ALK mutations and are used after crizotinib, and some of them have even replaced crizotinib as first-line option among patients with ALK-rearranged NSCLC [17–19]. Second generation ALKi ceritinib, alectinib and brigatinib have been approved for the treatment of ALK+ NSCLC patients after treatment with crizotinib (ceritinib in 2014 [20] and in 2015 [21]; alectinib in 2015 [22] and in 2017 [23], while brigatinib in 2017 [24] and 2018 [25] for the United States and Europe, respectively) and as first-line TKI treatments (in 2017, ceritinib [26, 27] and alectinib [23, 28] and, in 2020, brigatinib [25, 29], for the United States and Europe, respectively).

At the time of the design and performance of the study, clinical trials had provided promising results for both brigatinib and alectinib as first-line TKIs in TKI-naïve ALK+ NSCLC patients, compared to crizotinib. The ALEX phase III study (https://clinicaltrials.gov/ number, NCT02075840), showed that alectinib had a superior investigator-assessed PFS versus crizotinib (HR, 0.47; *P* < 0.001 [30]). At the second interim analysis of the ALTA-1L phase III trial (https://clinicaltrials.gov/ number, NCT02737501) the blinded independent review committee (BIRC)-assessed HR of PFS was 0.49 (log rank *P* < 0.0001) [31]. Moreover, both drugs present relevant intracranial efficacy: alectinib demonstrated superior efficacy versus crizotinib regardless of baseline CNS metastases [32] and brigatinib significantly delayed both CNS progression (without prior systemic progression) and systemic progression (without prior intracranial progression) compared with crizotinib [33]. Regarding ceritinib, direct comparison in first line has only been performed with chemotherapy [34, 35], although indirect comparison showed better results for ceritinib than crizotinib [36]. At the time of the beginning of the current study, two other ALK inhibitors were being tested in first line in comparison to crizotinib, although no results were available (ensartinib in eXalt3, NCT02767804, or lorlatinib in CROWN, NCT03052608).

Although no direct comparison between alectinib, brigatinib and ceritinib has been performed in a first-line setting, there are indirect comparisons in second line from which hypotheses can be drawn. Ceritinib, alectinib and brigatinib are effective in crizotinib-refractory ALK+ NSCLC patients [37–39], but no direct comparison between these drugs after crizotinib is available. An ongoing trial, ALTA-3 (https://clinicaltrials.gov/ number, NCT03596866), compares the efficacy of alectinib versus brigatinib in ALK+ NSCLC patients who had progressed on crizotinib; besides, and according to the current lack of direct comparisons, indirect analyses using available data have been performed to compare them. In fact, a matching-adjusted indirect comparison (MAIC) [40] between these drugs in crizotinib-refractory ALK+ NSCLC patients (using clinical data from the ALTA trial – date February 21, 2017 –, ASCEND-1 [41], ASCEND-2 [42], NP28761 [43] and NP28673 [44]) suggested that brigatinib may have prolonged PFS and OS versus ceritinib and prolonged PFS versus alectinib in patients after progression with crizotinib.

From a safety perspective, all ALKi are considered to be safe and tolerable in a similar fashion [45], although they show adverse events (AE), some of them common and others drug-specific. A systematic review [46] concluded that crizotinib was associated with more gastrointestinal and visual events, alectinib tended to have more hepatic and musculoskeletal AEs, ceritinib presented the highest incidence of clinically significant gastrointestinal AEs and laboratory abnormalities and brigatinib had a unique profile of increased early onset pulmonary AEs and hypertension associated with the 180 mg dose; these pulmonary AEs were found to be reduced when using the recommended initial dose of 90 mg [47]. This systematic review also suggested ceritinib to be less preferred by clinicians due to its safety profile. Regardless of their differences, most of the safety concerns associated with the mentioned ALKi can be minimized reducing administration dose [46].

According to first-line results with brigatinib and alectinib and indirect results of ceritinib, this last drug seems to have a lower efficacy both at systemic and cerebral levels when compared to brigatinib and alectinib [40]. As tolerability of all these ALKi is similar and alectinib has become the standard of care, a head to head clinical trial comparing brigatinib and alectinib as first-line therapy would be very interesting. However, since this head to head is not planned, results obtained with these second generation ALKi in the ALTA-2 study in second line, as well as in the MAIC analysis, will help to elucidate and refine the first-line therapy outline. Besides, *in silico* investigational approaches may be an alternative to compare the potential benefits of both drugs.

Concerning the mechanism of action of alectinib and brigatinib, both share ALK as a protein target, but they display completely different target profiles that could be determinant to define each drug mechanism. Beside ALK, brigatinib targets other tyrosine-protein kinases receptors such as EGFR [48–50], receptor-type tyrosine-protein kinase FLT3 [48, 51], tyrosine-protein kinase FER [52], ROS1 [51, 53], and insulin-like growth factor 1 receptor (IGF1R) [48, 49, 51, 54]. On the other hand, alectinib inhibits RET with comparable potency to ALK [55].

*In silico* tools are useful resources for predicting several (bio)chemical and (patho)physiological characteristics of likewise potential drugs [56]. These methods are used to improve *in vivo* and *in vitro* models and refine experimental programs of clinical and general biomedical studies involving lab work [57], and, in the long run, can reduce lab work and effectively succeed in 3R (reduce, reuse, recycle) [58]. Overall, these systems can be employed for the exploration of anticancer drug mechanisms of action and their efficacy in specific patient profiles.

In the present study, we created *in silico* systems biology-based mechanistic models of two first-line approved second generation ALKi, brigatinib and alectinib, in order to explore the potential differences between them with the aim of providing information or raising hypotheses towards the identification of strengths and weaknesses of the mechanisms of action of both drugs as first-line treatment for ALK+ NSCLC patients.

## RESULTS

**Figure 1:**
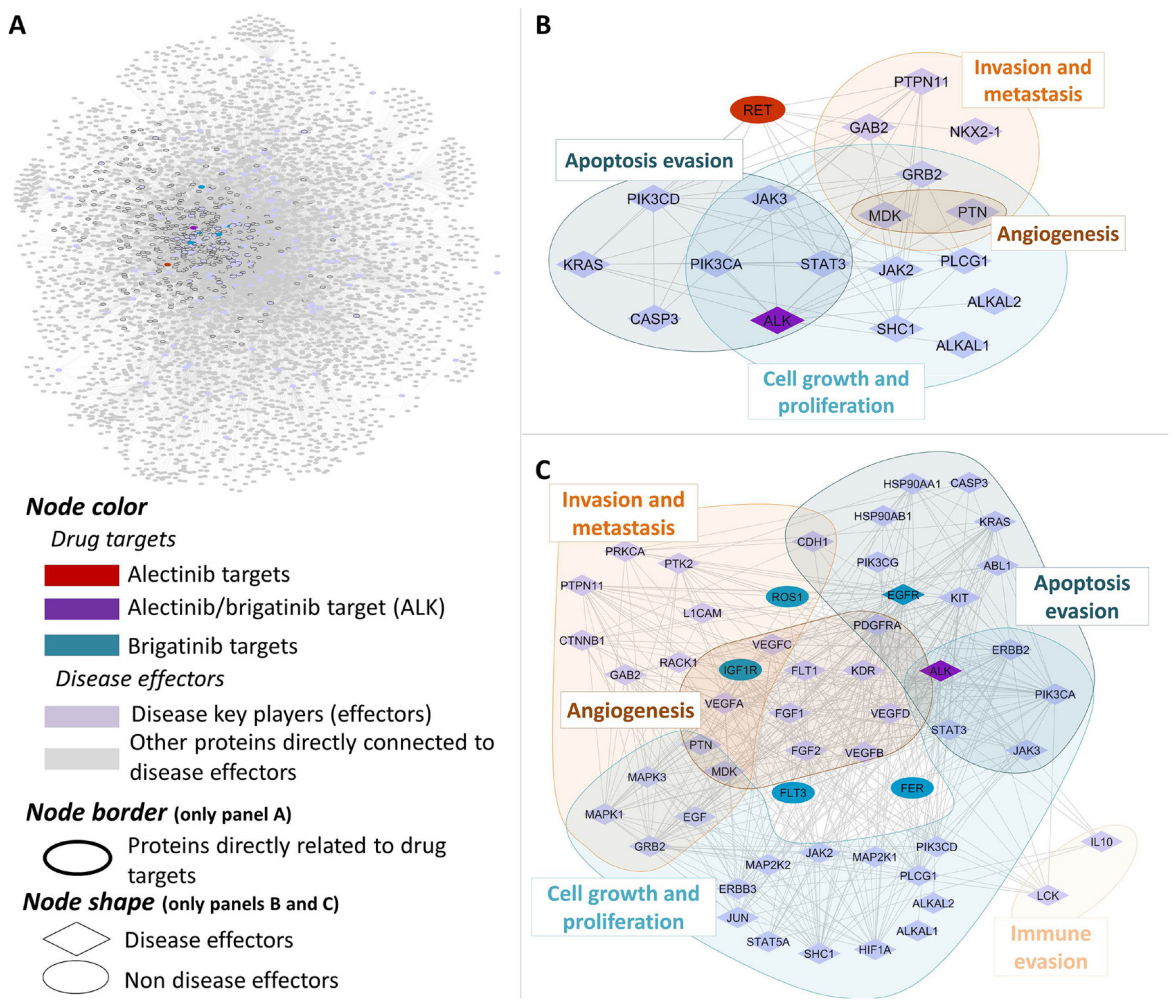
Human protein networks around ALK+ NSCLC molecular pathophysiology. General overview (**A**) and centered on main disease players (indicating their pathophysiological motives) and their relationship to alectinib (**B**) and brigatinib (**C**) drug targets.

The main pathophysiological processes (namely “motives”) described to be involved in ALK+ NSCLC were: (1) Cell growth and proliferation, (2) Sustained angiogenesis, (3) Evading apoptosis, (4) Tissue invasion and metastasis, (5) Immune evasion (Supplementary Table 1). Subsequently, each pathophysiological process was functionally characterized at protein level to determine its molecular effectors and used for focusing the analysis towards ALK+ NSCLC in a human biological network context (Figure 1 and Supplementary Table 2). Brigatinib and alectinib protein target profiles were also carefully characterized and used in the posterior analyses (Figure 1 and Supplementary Table 3). Mechanistic systems biology models of brigatinib and alectinib obtained with TPMS technology were constructed with accuracy values of 94% to evaluate their mechanism of action and potential treatment efficacy in ALK+ NSCLC. Two distinct modelling approaches were used for that purpose: Artificial neural networks (ANN) [59], with the aim of detecting biological relationships; and sampling-based methods [60], in order to explain those relationships. A Sobol sensibility analysis was applied to brigatinib and alectinib mechanistic models in order to evaluate their robustness. The results of this analysis are available in the Supplementary Methods.

### Effect of brigatinib and alectinib on cell growth, apoptosis and immune evasion through ALK and non-ALK inhibition

The relationships of each drug target with ALK+ NSCLC main pathophysiological motives were evaluated by the ANN and the results are shown in Table 1. The ANN analysis showed that, in general, alectinib presented a slightly lower correlation with ALK+ NSCLC pathophysiology than brigatinib (around 80% of the score obtained by brigatinib).

**Table 1:** Effect of brigatinib’s and alectinib’s drug targets in ALK+ NSCLC

Pathophysiological processes (motives)	Brigatinib’s targets						Alectinib’s targets	
	*ALK*	*FLT3*	*FER*	*ROS1*	*IGF1R*	*EGFR*	*ALK*	*RET*
NSCLC ALK^+^	++	+	+	+	+	+++	++	+
Cell growth and proliferation	+++	++	++	+	+++	+++	+++	+
Evading apoptosis	+++	++	+	+	++	++++	+++	+
Sustained angiogenesis	+	+	++	+	+++	+	+	+
Tissue invasion and metastasis	+	+	++	+	++	++	+	+
Immune evasion	+++	+++	+	+	+	++	+++	++++

Relationship of brigatinib’s and alectinib’s targets and pathophysiological motives calculated by artificial neural networks (ANN). High (++++) corresponds to *p*-value < 0.05, Medium-high (+++) corresponds to *p*-values < 0.15 and Medium (++) corresponds to *p*-value < 0.25, while Low (+) correspond to *p*-value > 0.25.

Evaluation of the relations between individual pathophysiological motives and drug targets suggested that both drugs affect cell growth and proliferation, apoptosis evasion and immune evasion through ALK inhibition. Regarding alectinib, its inhibition of RET might occur through modulation of the tumour immune response. On the other side, brigatinib non-ALK targets might affect the pathophysiological motives already affected by ALK inhibition (FLT3, IGF1R, and especially EGFR), as well as angiogenesis and invasiveness through FER and IGF1R inhibition.

### Brigatinib and alectinib non-ALK targets affect differently cancer-related processes, including proliferation, apoptosis evasion, invasiveness and immune evasion

**Figure 2:**
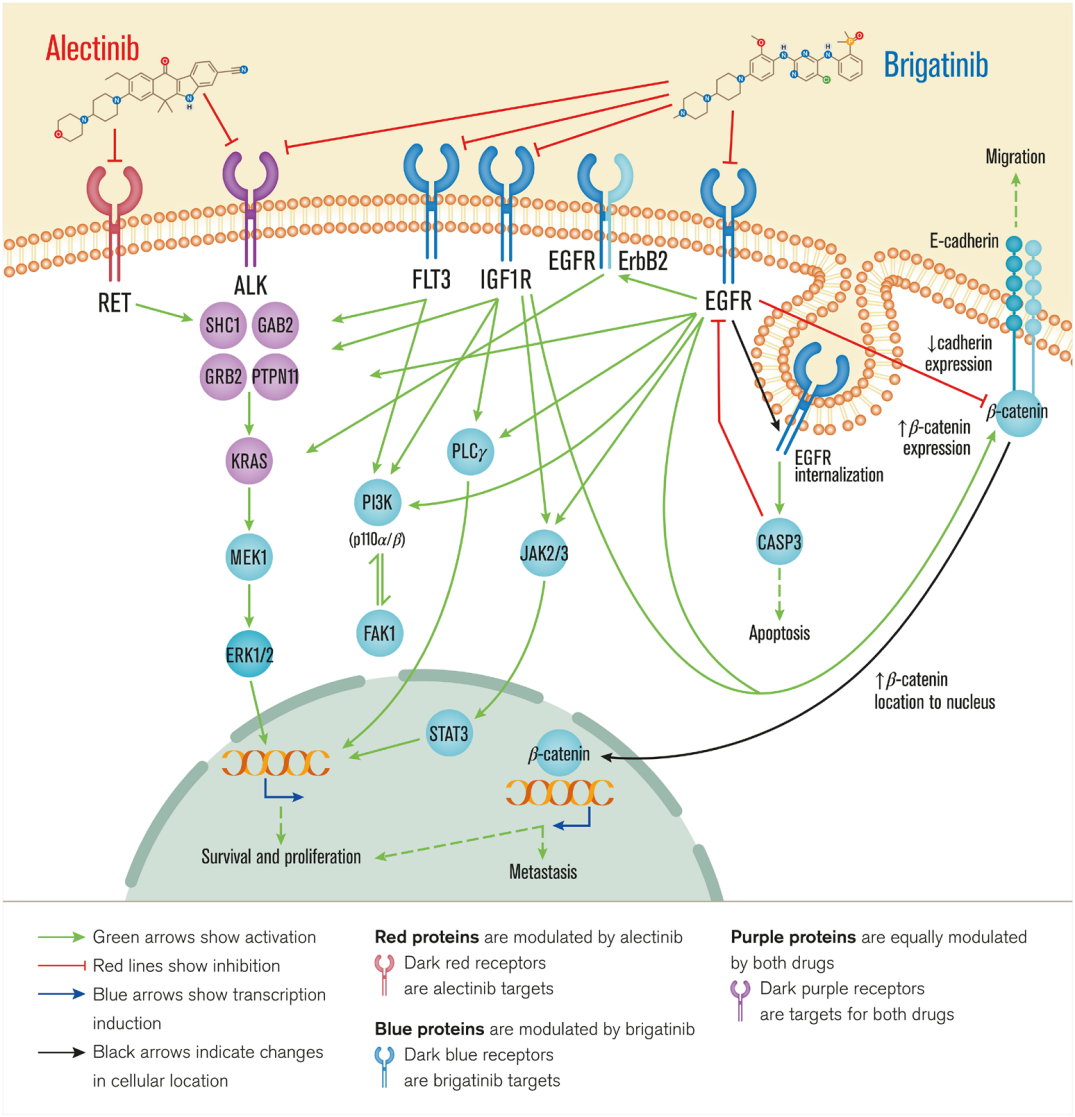
Overview of brigatinib’s and alectinib’s mechanisms of action. Receptor targets of each drug are depicted through the cell membrane and the following pathways and pathophysiological motives affected are depicted from the cell surface to the nucleus. Alectinib acts through ALK and RET, involved mainly in survival and proliferation, while brigatinib acts also through ALK and FLT3, IGF1R, and EGFR, signaling through overlapping intracellular mechanisms affecting cell survival and proliferation, metastasis, apoptosis and migration. Bibliographical validation information of interactions on the predicted mechanisms of action are shown in Supplementary data (Supplementary Table 1).

The comparison of the predicted mechanisms of action obtained by the mechanistic systems biology modeling using TPMS technology (Figure 2 and Supplementary Table 4) shows that both drugs act through ALK and some overlapping intracellular mechanisms (involving SHC1, GRB2, RASK). However, brigatinib seems to achieve a more diverse downstream effect, through PI3K, ERK and JAK/STAT.

**Figure 3:**
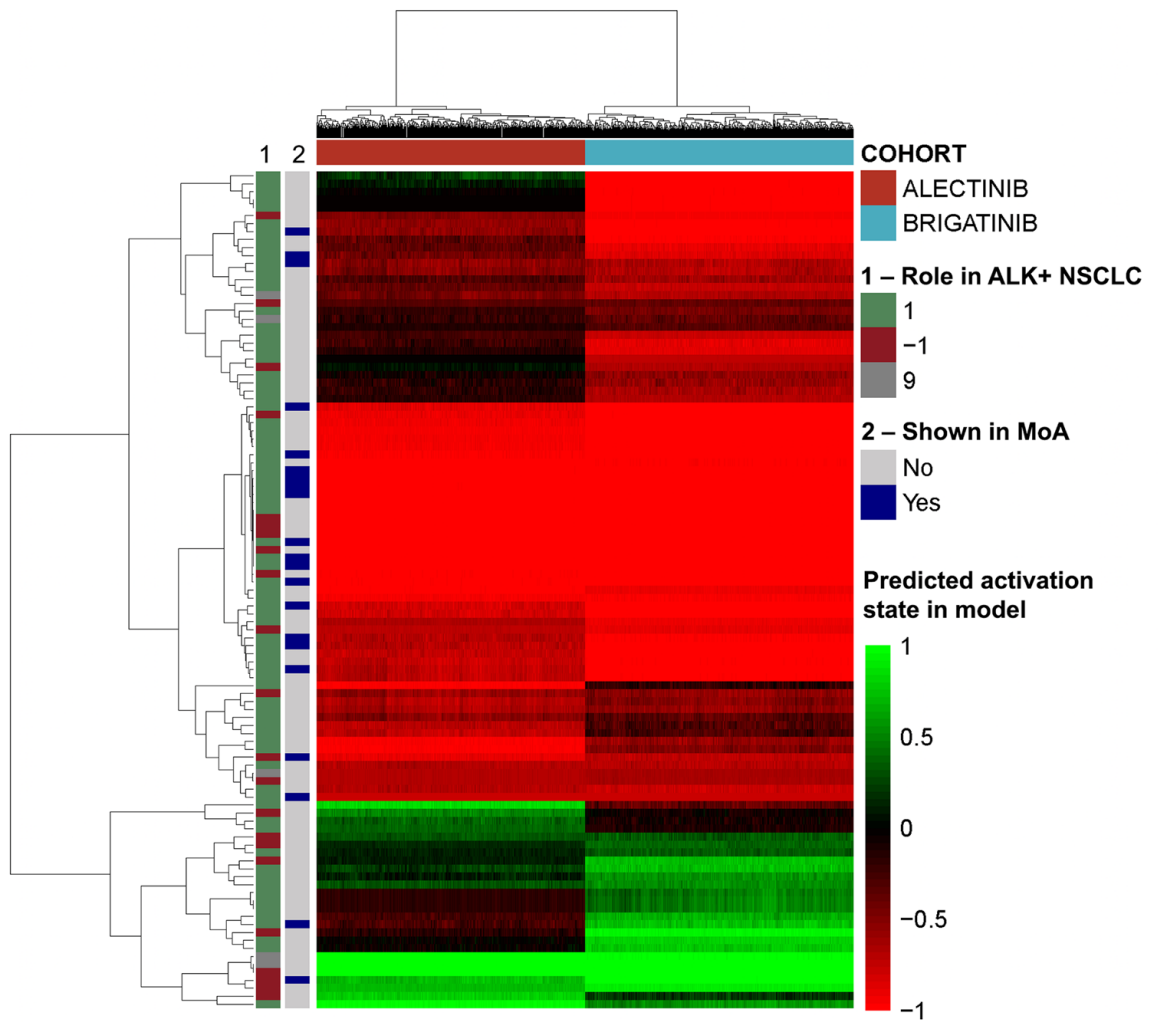
Heatmap of the effect induced by brigatinib and alectinib in each model solution over the effectors of the pathology. The vertical bars indicate the pathological effect of the effectors (1 if activated in the pathology, -1 if inhibited in the pathology, 9 if complex role) and whether the proteins appear in Figure 2 (“MoA”).

A further evaluation of the impact of each drug on the activity of each protein present in the mechanisms of action, and on the pathophysiological motives previously defined was carried out. This analysis showed that brigatinib, compared to alectinib, has a stronger effect (TSignal) on most of the proteins and all the motives defining ALK+ NSCLC (Figure 3) except in immune evasion (Table 2), for which alectinib presents a greater effect.

**Table 2:** Effect of brigatinib and alectinib over each pathophysiological motive measured by Tsignal

Motive	% Effectors more reversed		Drug with highest Tsignal	FDR *T*-Test
	Brigatinib MoA	Alectinib MoA		
Cell growth and proliferation	87%	10%	Brigatinib	< 0.05
Evading apoptosis	69%	27%	Brigatinib	< 0.05
Sustained angiogenesis	65%	29%	Brigatinib	< 0.05
Tissue invasion and metastasis	63%	37%	Brigatinib	< 0.05
Immune evasion	25%	75%	Alectinib	< 0.05

Percentage of effectors reversed indicate the proportion of proteins in each motive with a significant difference (FDR < 0.05) and stronger modulation considering the total number of effectors affected in the mechanism of action (MoA) for each drug.

### Effect of brigatinib and alectinib on invasiveness and central nervous system metastasis

As shown in Table 2, brigatinib was predicted to have a potential stronger effect on metastasis effectors, which are related to invasiveness promotion and metastasis-site characteristics. In order to assess the possible role of each drug on brain metastasis, eight protein/gene effectors known to have a more important role in brain metastasis than in primary tumours were considered: FGFR1 [61–63]; Ki-67 [64, 65], ROBO1 [66]; S100A7 [67, 68]; S100B [69, 70]; SIRT1 [71]; SLIT2 [72]; and VEGFA [61, 67]. Out of these, six (Ki-67, ROBO1, S100A7, S100B, SLIT2, VEGFA) were found to be significantly more inhibited by brigatinib than alectinib (FDR < 0.05 and a change in TSignal > 20%). The current analysis also showed an association between brigatinib and prevention of brain metastasis, mainly through EGFR and IGF1R.

### Susceptibility of brigatinib and alectinib to bypass resistance mechanisms

The impact of resistance mechanisms, via protein mutations, on brigatinib’s and alectinib’s mechanisms of action TPMS models was evaluated over a total of 935 proteins and 2805 modifications (activation, inhibition, deletion) (Supplementary Table 5). Of those, 55 different modifications were identified as potential treatment resistances for brigatinib and 93 for alectinib. Among them, 37 were shared between the two drugs. The potential resistance mechanisms that affected alectinib to a greater extent than brigatinib were, mostly, related to the alternative pro-proliferative signaling mechanisms by which NSCLC cells could continue to proliferate. These mechanisms included proteins like MET, ERBB, FGFR, NTRK1 or PDGFR, among others. On the other hand, the potential resistance mechanisms that affected either both drugs or brigatinib to a greater extent than alectinib were mainly potential downstream mediators such as SHC1, KRAS, PI3K or ERK.

### Effect of concomitant treatments on the mechanism of action of brigatinib and alectinib

The potential interference with brigatinib or alectinib mechanisms of action was evaluated using a total of 654 drugs (Supplementary Table 6). The drugs that may impact the brigatinib mechanism may include angiotensin-receptor blockers, barbiturates and bisphosphonates. This can be mitigated by adjusting the brigatinib dose. For alectinib, drugs such as non-peptide inhibitors of the antidiuretic hormone can also interfere with it. However, concomitant use of brigatinib with strong/moderate CYP3A inhibitors/inducers can be managed [72].

## DISCUSSION

The second generation ALKi brigatinib and alectinib have demonstrated efficacy in second line treatment in crizotinib-refractory ALK+ NSCLC patients, and in the first-line setting in ALEX (alectinib) and ALTA-1L (brigatinib) clinical trials. In the absence of a head to head trial between brigatinib and alectinib in the first-line setting, and beside the efficacy data and toxicity profile information obtained in independent trials, information from clinical trials in a second-line setting and indirect approaches may help to elucidate the best therapy against ALK+ NSCLC. In the present study, we applied *in silico* systems biology approaches to compare brigatinib and alectinib as first-line treatment for ALK+ NSCLC at a mechanistic level and thus highlighting the strengths and weaknesses of each ALK inhibitor. The present study indicates that both brigatinib and alectinib could be reasonable choices for first-line treatment, as also previously suggested by other authors [47]. The results obtained by our *in silico* model allow differentiating between the mechanisms of action of each drug, suggesting that both drugs may have similar efficacy as first-line treatment, and brigatinib may have higher impact in most studied pathways than alectinib. Other specific characteristics were highlighted for each drug.

According to previous publications [17, 47], brigatinib acts as a multi-kinase inhibitor with a broad-spectrum activity against ALK, FLT3, FER, ROS1, IGF1R, and EGFR targets, while alectinib acts on ALK and the proto-oncogene RET [55]. The analyses performed in this study to further determine the differences between brigatinib and alectinib’s mechanisms of action point towards a potentially relevant role of RET, EFGR, IGF1R and FLT3 (besides ALK) in treating NSCLC. All these targets had been previously related to a greater or lesser extent to NSCLC development [73–77].

As predicted by TPMS analyses (Figure 2), brigatinib targets appear to show a more diverse range of effects compared to alectinib, mediated by ALK inhibition on NSCLC, such as: cell growth and proliferation (as for example STAT3 [78], PI3K [79], K-Ras [78, 80] or erbB2 [81] signaling); evading apoptosis (through EGFR-CASP3 interplay [82]); acting over sustained angiogenesis (IGF1R signaling [83, 84]); and tissue invasion and metastasis processes (modulating the E-cadherin-β-catenin axis [85–88]). These predicted results and the observed broad range of different effects of brigatinib could be explained by a wider cancer-related target profile of brigatinib. Moreover, it could also be associated with the relatively longer PFS observed with brigatinib in the crizotinib-refractory setting as compared to alectinib [89]. On the other hand, the analyses also suggest that alectinib might have a greater effect on immune evasion regulation through RET inhibition.

Central nervous system (CNS) is one of the most common sites of first progression in ALK+ NSCLC [89]. Even while receiving crizotinib (in around 25–50% of cases), efficacy end points are lower in relation to the CNS than overall [90–92]. In our study, brigatinib was predicted to have a potentially more robust impact on brain metastasis effectors than alectinib. Inhibition of EGFR might prevent CADH1 reduction mediated by PI3K/FAK1 and thus inhibit tissue invasion. Blocking EGFR and IGF1R pathways might also prevent β-catenin (CTNB1) upregulation, accumulation in the nucleus and transcription factor function. Intracranial responses to TKIs have also been observed in previous studies. In the phase III trial ALTA-1L (https://clinicaltrials.gov/: NCT02737501) brigatinib was associated with a higher intracranial objective response rate (iORR) (78%) in individuals with ALK TKI-naive ALK+ NSCLC with baseline brain metastases compared to crizotinib (26%) [47]. Alectinib also showed superior intracranial activity versus crizotinib (81% and 50%, respectively) in the ALEX clinical trial, although progression in the brain with both agents has also been observed [93]. This intracranial efficacy is clearly explained by brain bioavailability in the case of alectinib, which shows a very good blood-brain barrier penetration [93], without being affected by MDR1/p-gp modulation [23, 94]. Brigatinib might be susceptible to MDR1/p-gp modulation [95], although no major concerns were raised by the regulatory bodies [25]. Thus, some other factors might explain brigatinib’s activity in the brain. The mechanistic study of the current analysis suggests that brigatinib might be able to reverse the activation of a greater percentage of metastasis effectors and, specifically, brain-related metastasis effectors, compared to alectinib. These effectors include the well-known proliferation marker Ki-67 [96] overexpressed in brain metastasis when compared to primary tumours [64, 65]; the ROBO1/SLIT2 axis, increased in brain metastasis [66] and involved in cell migration [97]; the pro-angiogenic VEGFA, related to increased brain metastatic potential [75, 98, 99]; and damage signal proteins (DAMPS) S100 proteins, involved in increased proliferation, anti-apoptotic, and migration capabilities [100, 101], which are increased in serum of brain metastatic patients and brain metastasis models [69, 70, 75, 100, 101]. Enhanced mechanistic impact over these - and other non brain-specific - metastasis effectors by brigatinib might explain its activity in the brain despite its lower blood-brain barrier penetration. The ongoing ALTA-3 trial will provide valuable information including intracranial progression after brigatinib versus alectinib in crizotinib-refractory patients that might help better understand their anti-metastatic mechanisms.

The acquisition of resistance to TKI therapy still seems inevitable. However, next generation TKIs are able to more strongly inhibit ALK – both in its wild type form and presenting secondary mutations – suggesting a better control over these progression mechanisms. In fact, brigatinib presents a high selectivity for ALK and low propensity for pharmacological failure [17, 19], showing higher potency than alectinib towards ALK-rearrangement fusions [14, 102, 103]. Brigatinib selectivity over ALK has been also proven in patients with ALK fusion proteins with and without secondary mutations [104, 105]. Little is known about the capacity of ALK TKIs to prevent bypass resistance mechanisms. The evaluation of the impact of developing non-ALK-related resistances on the efficacy of the drugs performed in the current study suggests that alectinib might be more susceptible to bypass resistance mechanisms. The results of our *in silico* analysis also suggest that brigatinib might block or prevent the development of upstream bypass resistance mechanisms more effectively than alectinib, which could translate into resistance-free treatment for a longer period of time. This would probably occur due to a mechanism of action that reaches a larger number of intracellular effectors involved in ALK-independent resistance mechanisms, including JAK/STAT, MEK/ERK, PI3K or PLCγ [105, 106]. According to our *in silico* results, brigatinib is predicted to modulate these pathways that are involved in different NSCLC-related pathophysiological processes, more strongly than alectinib. Thus, given the broader impact of brigatinib on ALK secondary mutations compared to other ALK TKIs [17, 19] and the results of the current analysis regarding bypass mechanisms, it could be hypothesized that brigatinib would prevent the generation of a wider spectrum of resistance mechanisms compared to alectinib. This low resistance predisposition of brigatinib could be related to the efficacy results in terms of PFS observed in the indirect comparison (MAIC) between brigatinib and alectinib/ceritinib by Reckamp [40]. However, further pre-clinical and clinical studies are needed to validate these hypotheses, and ALTA-3, comparing brigatinib to alectinib in ALK+ NSCLC patients who had progressed on crizotinib, might provide interesting conclusions in this regard.

There are two dimensions in which drugs can affect each other: through metabolic and mechanistic interactions. According to the recommendations of the technical specifications [23, 25], whereas both drugs interact with CYP3A – among other enzymes and transporters–, only brigatinib has strict interactions with the usage of inductors, inhibitors and substrates of CYP3A family cytochromes [25, 96]. The current study evaluated the mechanistic interaction between drugs commonly used in cancer patients, regarding the interference of the signal induced by the targets of co-treatments.

According to the current knowledge and the data herein presented, brigatinib might be more prone to present relevant metabolic and mechanistic interactions with other drugs than alectinib, which might be a safer option in poly-treated patients. Use of more than one drug (e.g., to treat cancer or treatment-derived complications, or pre-existing conditions) is common in cancer patients, and polypharmacy (5 or more concomitant drugs) has been shown to occur at a higher frequency in cancer survivors than in non-cancer age- and sex-matched controls [107]. Polypharmacy is especially common among the elderly or in end-of-life settings [108]. Thus, drug interactions must be carefully taken into account when considering different treatment options. However, as NSCLC adenocarcinoma patients tend to be younger and tend to be non-smokers compared to other cancer patients [102, 109], potential drug interference due to polypharmacy might not represent a determinant factor for treatment selection in clinical practice.

As previously stated, ALKi activity is affected by several factors, including tumour intrinsic characteristics (e.g., ALK fusion gene variants or presence of other primary gene co-mutations) and extrinsic factors (e.g., impact of prior treatments such as presence of ALK secondary mutations, or development of by-pass resistances), and also drug-dependent characteristics (e.g., blood-brain barrier crossing).

The current study aimed to explore mechanistic differences between brigatinib and alectinib that could affect efficacy of both drugs in an *in silico* approach. However, beside efficacy data, drug toxicity profile is an important determinant of treatment selection. According to previous publications, we considered that although all ALKi present common and specific adverse events, alectinib and brigatinib are similarly well tolerated and can be managed by reducing dose or interrupting treatment [46].

In order to better contextualize the hypotheses raised from the mechanistic analyses, other parameters need to be considered and have been herein discussed (ALK secondary mutations, safety concerns), and must be taken into account in the clinical practice. Besides, *in silico* modelling approaches can be used as predictive tools and hypothesis generators, limited by the information about diseases and drugs. For example, unknown targets or not yet described pathophysiological processes might have a role in the mechanisms of action of the evaluated drugs. Nevertheless, the models were built by considering the whole human protein network and a wide range of drug-pathology relationships (Supplementary Table 7) [60], not only limited to NSCLC or oncologic indications, and they present cross-validation accuracies above 80% in the case of ANN models and above 90% in sampling methods-based models. Thus, even if modelling approaches based on systems biology are limited by the amount of available information and some assumptions have to be made, *in silico* techniques are helpful for understanding fundamental processes in cancer [110, 111]. These approaches allow us to explore investigational or marketed drugs with reduced experimental cost and in different settings. This proves to be especially important if clinical investigations are not going to be done soon or are complex to be conducted, as in the case of the brigatinib versus alectinib head to head study in a first-line setting. Similarly, a comparison to other second and third generation ALKi that have recently shown benefit with respect to crizotinib in the first line setting (ensartinib in eXalt3, NCT02767804, or lorlatinib in CROWN, NCT03052608) could provide further insights into the mechanisms behind ALK+NSCLC treatment. Thus, systems biology and artificial intelligence approaches can contribute to exploring unanswered questions and this may guide the development of ALK TKIs and the identification of the optimal treatment sequence in ALK+ NSCLC patients. Further *in silico* studies with the aim of identifying the best treatment sequence after brigatinib are ongoing.

## MATERIALS AND METHODS

### Molecular characterization of ALK+ NSCLC pathophysiology and drugs

To carefully characterize the pathophysiology of ALK+ NSCLC, we conducted an extensive and detailed full-length review of relevant review articles over the last 5 years in the PubMed database (from December 3rd 2013 to December 3rd 2018) using the following search string: ((“*ALK+-positive*” [TITLE] or “*ALK+*” [TITLE]) and (“*Non-Small Cell Lung Cancer*” [TITLE] or “*NSCLC*” [TITLE]) AND (“*MOLECULAR*” [TITLE/ABSTRACT] or “*PATHOGENESIS*” [TITLE/ABSTRACT] or “*PATHOPHYSIOLOGY*” [TITLE/ABSTRACT]) and *Review*[ptyp]) and ((“*Non-Small Cell Lung Cancer*” [TITLE] or “*NSCLC*” [TITLE]) AND (“*MOLECULAR*” [TITLE/ABSTRACT] or “*PATHOGENESIS*” [TITLE/ABSTRACT] or “*PATHOPHYSIOLOGY*” [TITLE/ABSTRACT]) and *Review*[ptyp]). The search was also expanded using article reference lists. The main pathophysiological processes (motives) described to be involved in ALK+ NSCLC were identified (Supplementary Table 1). Subsequently, each motive was further functionally characterized at protein level to determine its molecular effectors. A total of 174 proteins were identified (Supplementary Table 2).

For drug protein target profile definition (brigatinib and alectinib), a dedicated review of databases (DrugBank [112], STITCH [113], SuperTarget [114]) and of scientific literature was performed (Supplementary Table 3).

### TPMS technology: systems biology-based model creation

**Figure 4:**
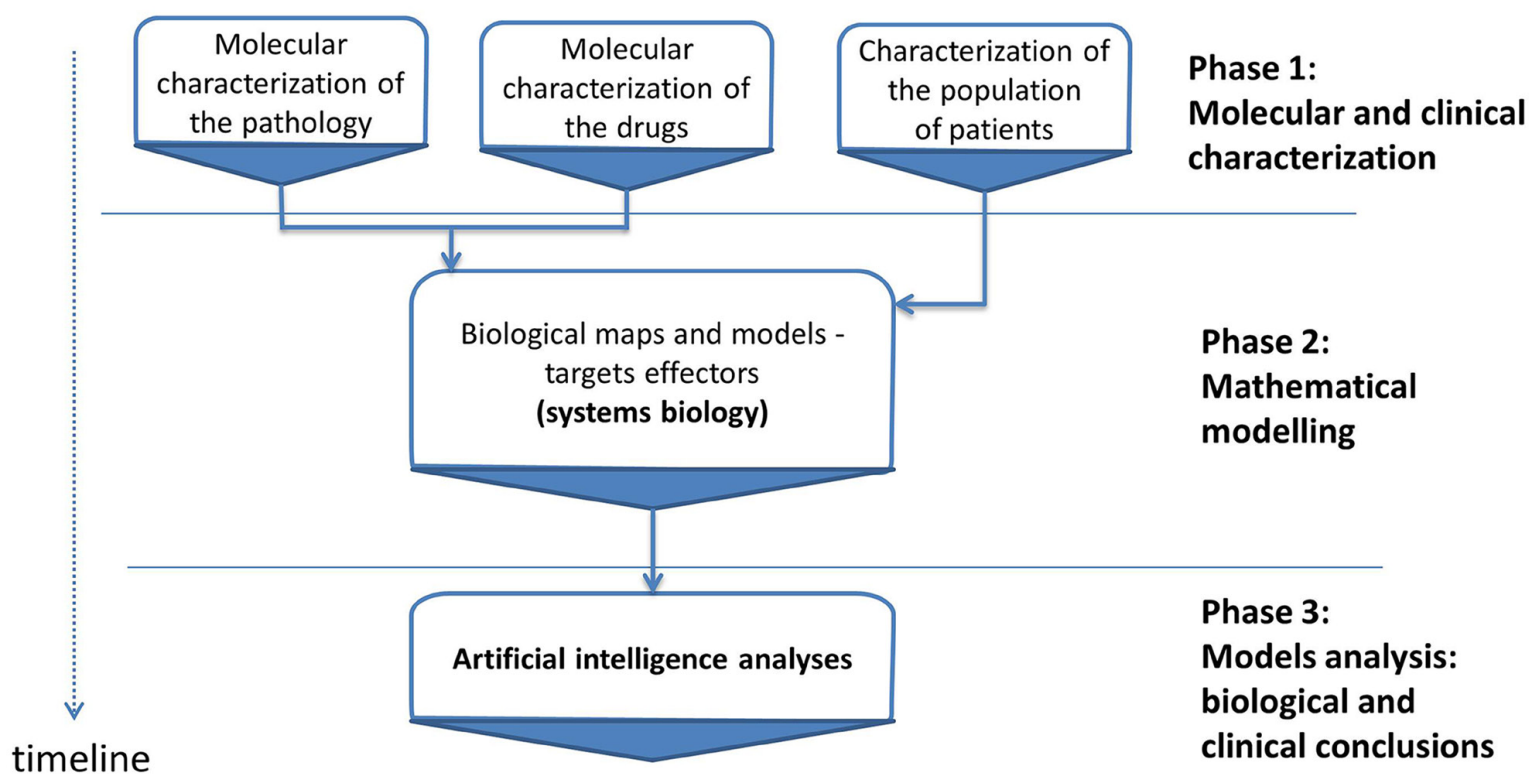
Study workflow. Overview of the *in silico* study approach showing the main phases employed to simulate the mechanisms of action (MoA) of brigatinib and alectinib with respect to ALK+NSCLC molecular characterization. TPMS is a validated top-down systems biology approach that integrates all available biological, pharmacological and medical knowledge (protein network, truth table and specific data compilation) by means of pattern recognition models and artificial intelligence to create mathematical models that simulate *in silico* the behavior of human physiology.

Therapeutic Performance Mapping System (TPMS) (Anaxomics Biotech, Barcelona, Spain) is a top-down systems biology approach based on artificial intelligence and pattern recognition models. This methodology integrates available biological, pharmacological and medical information to generate mathematical models that simulate the mechanisms of action of drugs in a pathophysiological human context (Figure 4). TPMS models are trained using a compendium of biological and clinical data characteristics of the human physiology (Supplementary Table 7).

### Mechanism of action models

In order to obtain the mechanism of action (MoA) of brigatinib and alectinib, drug-ALK+ NSCLC mathematical models were generated following the same methodology as described in Jorba [60] and applied in previous studies [63, 115, 116]. As input, TPMS takes the activation (+1) and inactivation (–1) of the drug target proteins (Supplementary Table 3), and as output the protein states of the pathology of interest (Supplementary Table 2). It then optimizes the paths between both protein sets and computes the activation and inactivation values of the full human interactome. The resulting subnetwork of proteins with non-null outputs and their values will define the MoA of the drug. The impact of each drug over the activity of the proteins effectors of the pathophysiological disease was quantified using the Tsignal (i.e., the average signal values of the protein effectors), as described in Jorba [60]. More detailed information on the modelling methodology can be found in Supplementary Methods.

### Sobol sensibility analysis

In order to analyze the impact of the noise in the final MoAs affecting the biological conclusions reached, a Sobol sensibility analysis was performed over the constructed TPMS mathematical models [117]. Detailed of the analysis implementation can be found in Supplementary Methods.

### Drug-(patho)physiology motive relation finding

Artificial neural networks (ANN) were used to identify relations between proteins (e.g., drug targets) and clinical elements of the network [118], which is an approach previously used and validated by several publications [53, 119–121]. This strategy was used to perform an efficacy evaluation of brigatinib and alectinib from each of their targets towards ALK+ NSCLC pathophysiological motives and its corresponding proteins. Detailed information on the modelling methodology can be found in Supplementary Methods.

### Evaluation of the impact of potential resistances over the mechanisms of action

In order to identify possible cancer resistances, the TPMS models were evaluated for possible mutations to identify the key nodes or proteins with higher impact on the effector proteins TSignal. Because both brigatinib and alectinib mechanisms of action had a vast amount of proteins or nodes (> 5000), the universe of possible key nodes was reduced to the list of proteins around ALK+ NSCLC effectors and around the drugs’ target proteins. To evaluate the addition of a mutation in the system, the impact of a protein activation, inhibition and deletion over the mechanisms of action of brigatinib and alectinib was tested. To do so, the resulting TSignal of the altered models was computed and compared to the original one. Finally, the *p*-value of the difference between the Tsignals over ALK+ NSCLC with and without the mutation was calculated, and the ones with *p*-values ≤ 0.022 were selected (Supplementary Table 5).

### Evaluation of drug interferences over the mechanisms of action

To identify possible co-treatment interferences, a list of pharmacological treatments potentially co-administered with brigatinib or alectinib was created and evaluated. To do so, we generated a list of all treatments for common conditions (either in the general population and in the ALK+ NSCLC population) and treatments for brigatinib/alectinib-associated adverse drug reactions, according to DrugBank database [112]. After that, the mechanisms of action of brigatinib and alectinib was perturbed by activating the co-treatment protein targets, each drug one by one, and the TSignal was computed. Finally, the differences in the TSignal between the original and the perturbed system and the corresponding *p*-values were calculated, and the ones with *p*-values < 0.1 and 0.05 were selected (Supplementary Table 6).

## CONCLUSIONS

An *in silico* head to head based on the mechanism of action evaluation between brigatinib and alectinib has been performed highlighting the advantages of using one before the other from an efficacy point of view. Brigatinib appears to have a wider mechanism of action, presenting targets that potentially act more strongly in most of the ALK+ NSCLC pathophysiological pathways, including invasiveness to the CNS. On the other side, alectinib-induced RET inhibition might contribute to reducing the tumour immune evasion mechanisms. In general, both drugs are known to be well-tolerated and, although shown and predicted to have a similar efficacy for the treatment of ALK+ NSCLC in a first-line setting, the differences in their target profiles might allow for identification, in subsequent studies, of different patient profiles that might benefit from either of them, beside considering potential safety concerns in specific patient subpopulations. Future clinical studies will be needed to confirm these findings. The used approach can be applied for the evaluation of other next-generation ALKi, even if not yet approved, or exploring other questions, such as optimal treatment sequence.

## SUPPLEMENTARY MATERIALS




