# Supplementary material for: Head to head evaluation of second generation ALK inhibitors brigatinib and alectinib as first-line treatment for ALK+ NSCLC using an in silico systems biology-based approach
Source: Oncotarget. 2021 Feb 16;12(4):316–32. doi: 10.18632/oncotarget.27875 (PMC7899557; doi:10.18632/oncotarget.27875)
Supplement: Supplementary file 1 [file oncotarget-12-316-s001.pdf]

Head to head evaluation of second generation ALK inhibitors brigatinib and alectinib as first-line treatment for ALK+ NSCLC using an *in silico* systems biology-based approach

SUPPLEMENTARY MATERIALS

TPMS technology: systems biology-based model creation and analysis for ALK+ NSCLC

Systems biology-based models were created using the Therapeutic Performance Mapping System (TPMS) to investigate the molecular Mechanisms of Action (MoA) of brigatinib and alectinib towards the modulation of ALK+ NSCLC. TPMS is a validated top-down systems biology approach that integrates all available biological, pharmacological and medical knowledge by means of pattern recognition models and artificial intelligence to create mathematical models that simulate in silico the behavior of human physiology. The methodology employed and detailed herein has been previously described [60] and applied elsewhere [59, 115, 116, 121]. Biological maps were transformed into a mathematical model capable of both reproducing existing knowledge and predicting new data. TPMS technology uses a set of artificial intelligence algorithms to generate the human physiology over the human biological network [121–124].

Human protein network (HPN) and truth table construction

A human protein network (HPN) was created in order to obtain the MoAs by including information from many public and private databases (KEGG [125, 126], REACTOME, [127] INTACT, [128] BIOGRID, [129] HPRD, [130] and TRRUST [131] and information extracted from scientific literature.

For the construction of the truth table, a selected collection of known input-output physiological signals considered the "truths" were collated into a table (Supplementary Table 7) and was used for training the models [132]. The truth table was based on a compendium of different databases that contain biological and clinical data [133, 134] and provides biological and pharmacological input-output relationships (such as drug-indication pairs). Information relating biological processes (adverse drug reactions, indications, diseases and molecular pathways) to their molecular effectors, i.e., each one of the proteins involved in the physiological process, was extracted from the biological effectors database (BED) (Anaxomics Biotech SL, [60]). The biological or pathological conditions under study were also included in the truth table and molecularly characterized through specific scientific literature search and hand-curated assignment of proteins to the conditions (Supplementary Table 2). The obtained final models had to be able to reproduce every rule contained in the truth table, and we defined the error of a model as the percentage of all the rules with which the model does not comply, while the accuracy was defined as the percentage of all the rules complied with.

Modelling strategies

Two complementary modelling strategies were used, (a) TPMS Artificial Neural Networks (ANNs) [59] and (b) TPMS Sampling-based Methods [60], to compare the efficacy of the drugs (defined as their targets, see Supplementary Table 3) and to compute the MoA models.

(a) ANNs are supervised algorithms that identify relations between proteins (e.g., drug targets) and clinical elements of a protein network [59, 118, 120, 135, 136] by inferring the probability of the existence of a specific relationship between two or more protein sets, based on the validation of the predictive capacity of the model towards the truth table. The learning methodology used consisted in an architecture of stratified ensembles of neural networks as a model, trained with a gradient descent algorithm to approximate the values of the given truth table. The neural network model used consisted in a Multilayer Perceptron (MLP) neural network classifier. MLP gradient descent training depends on randomization initialization and to avoid random errors 1000 MLPs are trained with the training subset and the best 100 MLPs are used. In order to correctly predict the effect of a drug independently of the number of targets, a different ensemble of neural networks are trained for a different subset of drugs according to their number of targets (drugs with 1 target, 2 targets, 3 targets). Then, the predictions for a query drug are calculated by all the ensembles, and pondered according to the number of targets of the query drug (the difference between the number of targets of the query and the number of targets of the drugs used to calculate each ensemble is used to ponder the result of each ensemble). A cross-validation with the truth table information showed that the accuracy of the described ANNs to reproduce the indications compiled in DrugBank

[112, 133] is 81.7% for those drugs with all targets in the human biological network.

(b) Sampling-based methods generate models similar to a MLP over the previously constructed HPN, where neurons are the proteins and the edges of the network are used to transfer the information (Supplementary Figure 1) [60]. This methodology was used for describing with high capability all plausible relationships between an input (or stimulus) and an output (or response). Sampling-based methods use optimization algorithms [123] to solve each parameter of the equation, i.e. the weights associated to the links between the nodes in the human protein network. In this approach, the network is limited by considering only interactions that connect drug targets with protein effectors in a maximum of three steps. The values of activation (+1) and inactivation (–1) of the protein targets of the drugs in the truth table were considered as input signals whereas the output is defined as the values of activation and inactivation of the proteins describing the phenotype (as retrieved from the BED). Each node of the protein network receives as input the output of the connected nodes in the direction flow from targets to effectors, weighted by each link weight (Supplementary Figure 1). The sum of inputs is transformed by a hyperbolic tangent function to generate the score of the node (neuron), which becomes the 'output signal' of the current node towards the nodes. The weight parameters are obtained by Stochastic Optimization Method based on Simulated Annealing [123], which uses probabilistic measures derived from the biological evidence to adjust network interaction types and strengths. Since the number of entries in the truth table is always smaller than the number of parameters (link weights) required by the algorithm, any process modelled by TPMS considers a population of different solutions.

Mechanisms of action elucidation

The MoAs obtained with the TPMS simulates potential interactions between drug targets and protein effectors associated to prototype-ALK+ NSCLC patients. In order to validate this approach, the intensity of the model's response, divided in TSignal and number of protein effectors activated, was used to understand the relationships between all potential mechanisms and compare sets of MoAs from different views (Supplementary Figure 1) [60].

Intensity of the response

We defined the "intensity" of the response as follows: 1) the quantity of protein effectors (#) that reach an expected signal sign; and 2) the strength or amount of the output signal reaching the effectors (i.e., a global measure of the output signal, named TSignal).

Given a protein effector "i", which reaches a signal value y_i, and v_i being the effector sign according to the BED (active or inactive) and n is the total number of effectors described for a phenotype, it was determined:

Number of effectors achieving the expected sign

Assuming that a drug may be able to activate/inactivate protein effectors reverting a disease/indication model phenotype. Using Dirac's δ (i.e. δ(0) = 1, and zero otherwise), the equation to calculate number of effectors achieving the expected sign for drug indications was defined as:

$$\#_{indication} = \sum_{i=1}^{n} \delta\left(v_i + \frac{y_i}{|y_i|}\right) \quad \text{[Equation 1]}$$

TSignal

The average output values of the protein effectors. For each effector, it was counted as positive signal if the sign is correct, and negative otherwise. When a drug affects a disease phenotype, v_i and y_i have opposite sign and it is necessary to change the sign in the corresponding equation:

$$TSignal_{indication} = -\frac{1}{n}\sum_{i=1}^{n} v_i y_i \quad \text{[Equation 2]}$$

Sobol sensitivity analysis

An adapted methodology for ensembles of high dimensional algorithms was applied following the definition of Sobol Sensitive Analysis [117]. According to the Sobol terminology, TPMS models can be redefined as follows:

$TSignal = TPMS\ (X)$ for $X = \{X_1, X_2 \dots X_n\}$ [Equation 3A and 3B]

Where x_i is each of the parameters used in the TPMS models. Then, the variation of TSignal for each x_i parameter can be expressed as:

$$\frac{d\,TSignal}{dX_i} = \frac{d\,TPMS(X)}{dX_i} \quad \text{[Equation 4A]}$$

Consequently, the variation of the simultaneous parameters x_i and x_j can be estimated as:

$$\frac{d\,TSignal}{dX_i\,dX_j} = \frac{d\,TPMS(X)}{dX_i\,dX_j} \quad \text{[Equation 4B]}$$

Using the previous equation descriptions, we measured the impact of varying random parameters over output TSignal in two different approaches, those being local analysis and global analysis [117].

Local sensitivity analysis

Local sensitivity analysis evaluates changes in the model outputs (TSignal) with respect to variations in a single model parameter. This effect was measured in the TPMS-models for both alectinib and brigatinib MoA models (Supplementary Figure 2).

Global sensitivity analysis

In the global sensitivity analysis, all parameters are varied simultaneously over the entire parameter space to measure the effects of their interactions on the model output. Given the high dimensionality of the TPMS ensemble models, this measure has been estimated by a MonteCarlo experiment to introduce random values (noise) in sets of 1200 candidate parameters. These final TSignal effects were measured by altering combinations of parameters in subsets of 1, 2, 3, 4, 5, 10, 15, 20 and 30 parameters simultaneously, from the candidate parameters list (Supplementary Figure 3).

Sensitivity results

Although TPMS-models have about 5000 parameters, only a small percentage of them showed a real impact on the output, which was less notorious in brigatinib than alectinib (Supplementary Figures 2 and 3). Nevertheless, the impact of some of the protein parameters are of great importance, meaning that TPMS models had to carefully adjust to all the restrictions defines in the truth table, while completing the drug-pathology model. We can see this as most protein parameters are actually part of the ALK+ NSCLC effectors (like P27361, P28482 and P414921, among others), which will definitely have a huge effect on the final TSignal according to its definition in Equation 2.

SUPPLEMENTARY REFERENCES

122. Kirkpatrick S. Optimization by Simulated Annealing Optimization by Simulated Annealing. Science. 1983; 220:671–80.

123. Rennard P, Collet JP. Stochastic Optimization Algorithms. Intelligent Information Technologies: Concepts, Methodologies, Tools, and Applications. 2011; 1121–1137.

124. Russell SJ, Norvig P. Artificial Intelligence A Modern Approach; (2nd. ed.). Pearson Education. 2003; 1151. https://doi.org/10.1017/S0269888900007724.

125. Kanehisa M, Susumu G. KEGG: Kyoto Encyclopedia of Genes and Genomes. Nucleic Acids Res. 2000; 28:27–30. https://doi.org/10.1093/nar/28.1.27. [PubMed]

126. Kanehisa M, Furumichi M, Tanabe M, Sato Y, Morishima K. KEGG: New perspectives on genomes, pathways, diseases and drugs. Nucleic Acids Res. 2017; 45:D353–61. https://doi.org/10.1093/nar/gkw1092. [PubMed]

127. Croft D, Mundo AF, Haw R, Milacic M, Weiser J, Wu G, Caudy M, Garapati P, Gillespie M, Kamdar MR, Jassal B, Jupe S, Matthews L, et al. The Reactome pathway knowledgebase. Nucleic Acids Res. 2014; 42:472–7. https://doi.org/10.1093/nar/gkt1102. [PubMed]

128. Orchard S, Ammari M, Aranda B, Breuza L, Briganti L, Broackes-Carter F, Campbell NH, Chavali G, Chen C, Del-Toro N, Duesbury M, Dumousseau M, Galeota E, et al. The MIntAct project - IntAct as a common curation platform for 11 molecular interaction databases. Nucleic Acids Res. 2014; 42:358–63. https://doi.org/10.1093/nar/gkt1115. [PubMed]

129. Salwinski L. The Database of Interacting Proteins: 2004 update. Nucleic Acids Res. 2004; 32:449D–451. https://doi.org/10.1093/nar/gkh086. [PubMed]

130. Keshava Prasad TS, Goel R, Kandasamy K, Keerthikumar S, Kumar S, Mathivanan S, Telikicherla D, Raju R, Shafreen B, Venugopal A, Balakrishnan L, Marimuthu A, Banerjee S, et al. Human Protein Reference Database-2009 update. Nucleic Acids Res. 2009; 37:767–72. https://doi.org/10.1093/nar/gkn892. [PubMed]

131. Han H, Shim H, Shin D, Shim JE, Ko Y, Shin J, Kim H, Cho A, Kim E, Lee T, Kim H, Kim K, Yang S, et al. TRRUST: A reference database of human transcriptional regulatory interactions. Sci Rep. 2015; 5:1–11. https://doi.org/10.1038/srep11432. [PubMed]

132. Anellis IH. Peirce's Truth-functional Analysis and the Origin of the Truth Table. Hist Philos Log. 2012; 33:87–97. https://doi.org/10.1080/01445340.2011.621702.

133. Wishart DS, Knox C, Guo AC, Cheng D, Shrivastava S, Tzur D, Gautam B, Hassanali M. DrugBank: A knowledgebase for drugs, drug actions and drug targets. Nucleic Acids Res. 2008; 36:901–6. https://doi.org/10.1093/nar/gkm958. [PubMed]

134. Kuhn M, Letunic I, Jensen LJ, Bork P. The SIDER database of drugs and side effects. Nucleic Acids Res. 2016; 44:D1075–9. https://doi.org/10.1093/nar/gkv1075. [PubMed]

135. Martín-Segura A, Casadomé-Perales Á, Fazzari P, Mas JM, Artigas L, Valls R, Nebreda AR, Dotti CG. Aging increases hippocampal DUSP2 by a membrane cholesterol loss-mediated RTK/p38MAPK activation mechanism. Front Neurol. 2019; 10:1–12. https://doi.org/10.3389/fneur.2019.00675. [PubMed]

136. Pujol A, Mosca R, Farrés J, Aloy P. Unveiling the role of network and systems biology in drug discovery. Trends Pharmacol Sci. 2010; 31:115–23. https://doi.org/10.1016/j.tips.2009.11.006. [PubMed]

Supplementary Table 1: Pathophysiological processes (motives) and number of seeds proteins of ALK+ NSCLC

MOTIVE #	Motive name	# proteins
1	CELL GROWTH AND PROLIFERATION	46
2	SUSTAINED ANGIOGENESIS	20
3	EVADING APOPTOSIS	50
4	TISSUE INVASION AND METASTASIS	73
5	IMMUNE EVASION	16

Supplementary Table 2: List of proteins used for ALK+ NSCLC molecular characterization. Motive number and protein state (1 for activation, -1 inhibition, - unknown) are included. See Supplementary Table 2

Supplementary Table 3: Brigatinib and alectinib characterized protein targets

Drug name	Uniprot ID	Gene Name	Effect	DrugBank candidate	Stitch candidate	Supertarget candidate	References
Alectinib	Q9UM73	ALK	-1	yes	yes	no	PMID: 28455243
Alectinib	P07949	RET	-1	no	yes	no	PMID: 25349307
Brigatinib	Q9UM73	ALK	-1	yes	yes	no	PMID: 2714483; 29075144
Brigatinib	P00533	EGFR*	-1	yes	no	no	PMID: 29451020; 29075144; 28287083
Brigatinib	P36888	FLT3	-1	yes	no	no	PMID: 27144831; 29451020
Brigatinib	P08922	ROS1	-1	no	no	no	PMID: 28680831; 29451020
Brigatinib	P16591	FER	-1	no	no	no	PMID: 29540831; FDA Multi-discipline review
Brigatinib	P08069	IGF1R	-1	yes	no	no	PMID: 27144831; 29451020; 29075144; 29403310

*Highest affinity for EGFR (L858R) mutated form.

Supplementary Table 4: Bibliographical validation of interactions on the predicted mechanisms of action. See Supplementary Table 4

Supplementary Table 5: List of proteins/modifications tested as alectinib/brigatinib resistance. See Supplementary Table 5

Supplementary Table 6: Drugs tested for co-treatment interference. See Supplementary Table 6

Supplementary Table 7: Summary of data used for model construction (Human Protein Network (HPN) and truth table)

Entry type		# Entries
**In-house databases information**		
	Considered Interactions	437.071
	Considered Proteins	16.961
	Characterized Drugs	5.414
	Drug Targets	2,690
	Characterized Clinical Conditions	253
	Clinical Conditions Key Proteins Characterized	4.076
**Truth table information**		
	Curated drug-indications restrictions	180,264 (1,731 positive)
	Drug-ADRs restrictions	30,096 (2,460 positive)
	Drug-indications/ADRs protein correlations	2.175

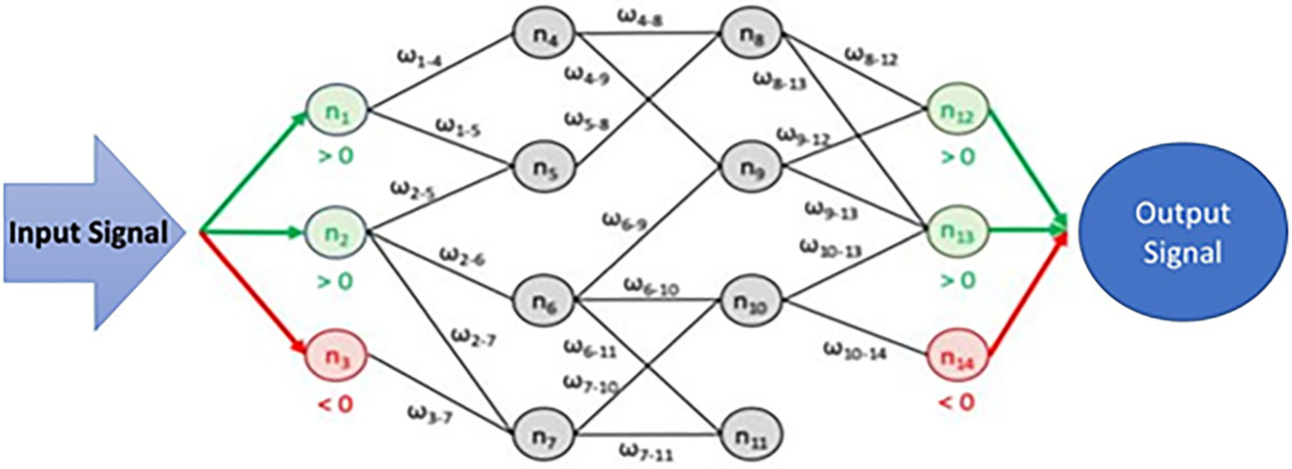

Supplementary Figure 1: TPMS schematic representation of the input/output signals information over the Human Protein Network (HPN) using a Multilayer Perceptron-like and sampling method to predict the Mechanisms of Action (MoAs) of a drug.

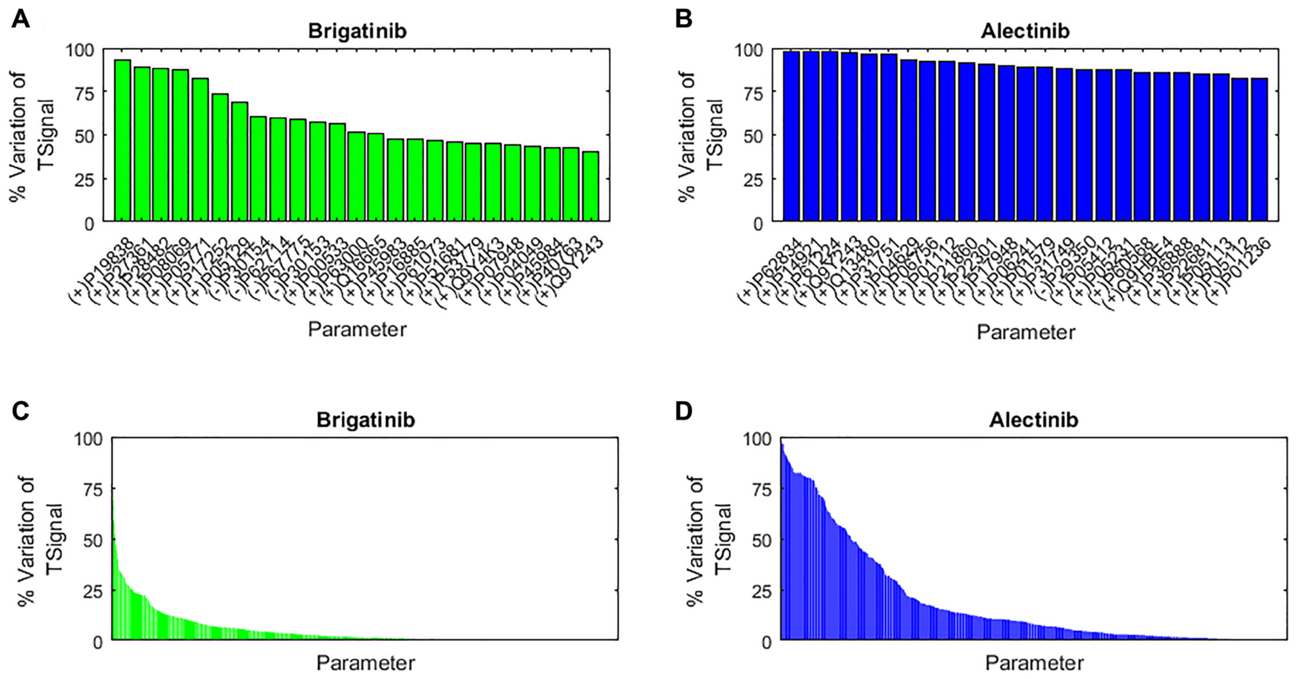

Supplementary Figure 2: MoA models difference of TSignal measured for each individual parameters variation. (**A** and **B**) show the % of variation of the output TSignal for the 25 most sensible parameters in brigatinib and alectinib MoA models, respectivelty. (**C** and **D**) show the % of variation of the output TSignal for all parameters in brigatinib and alectinib MoA models, respectively. Parameters are ordered from the ones affecting the most to the models, to the ones affecting the less. For the sake of visual simplicity, the parameter names of C and D are not displayed in the x axis.

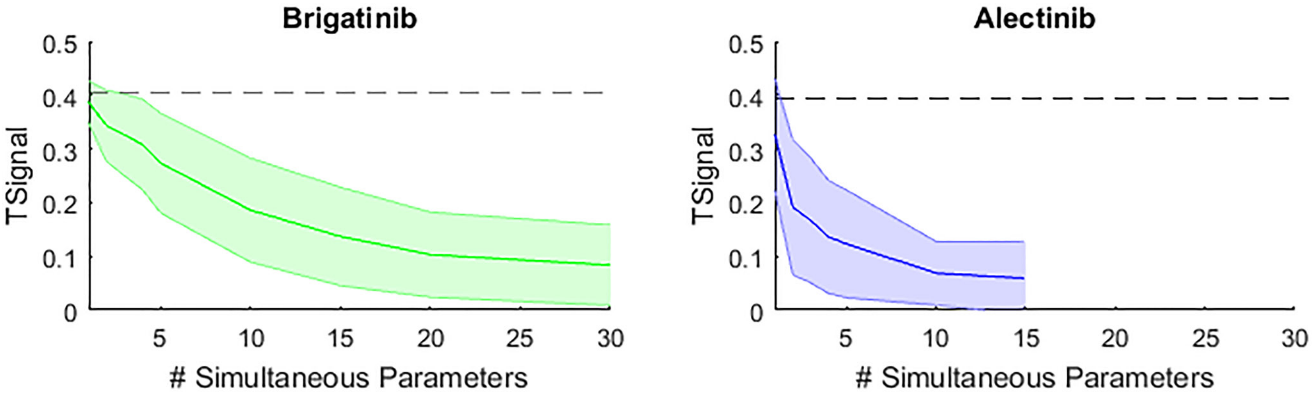

Supplementary Figure 3: MoA models TSignal measured for individual and multiple parameters variation. The output TSignal when variating 1 until 30 random parameters simultaneously in brigatinib and alectinib MoA models, respectively, is shown. The gray, dashed line represents the original TSignal values for each of the drugs MoA.
